# Mechanical Properties of Isolated Primary Cilia Measured by Micro-tensile Test and Atomic Force Microscopy

**DOI:** 10.3389/fbioe.2021.753805

**Published:** 2021-11-11

**Authors:** Tien-Dung Do, Jimuro Katsuyoshi, Haonai Cai, Toshiro Ohashi

**Affiliations:** ^1^ Division of Human Mechanical Systems and Design, Graduate School of Engineering, Hokkaido University, Sapporo, Japan; ^2^ Division of Mechanical and Aerospace Engineering, Faculty of Engineering, Hokkaido University, Sapporo, Japan

**Keywords:** isolated primary cilia, young’s modulus, viscoelasticity, micro-tensile test, AFM test

## Abstract

Mechanotransduction is a well-known mechanism by which cells sense their surrounding mechanical environment, convert mechanical stimuli into biochemical signals, and eventually change their morphology and functions. Primary cilia are believed to be mechanosensors existing on the surface of the cell membrane and support cells to sense surrounding mechanical signals. Knowing the mechanical properties of primary cilia is essential to understand their responses, such as sensitivity to mechanical stimuli. Previous studies have so far conducted flow experiments or optical trap techniques to measure the flexural rigidity *EI* (*E*: Young’s modulus, *I*: second moment of inertia) of primary cilia; however, the flexural rigidity is not a material property of materials and depends on mathematical models used in the determination, leading to a discrepancy between studies. For better characterization of primary cilia mechanics, Young’s modulus should be directly and precisely measured. In this study, the tensile Young’s modulus of isolated primary cilia is, for the first time, measured by using an in-house micro-tensile tester. The different strain rates of 0.01–0.3 s^−1^ were applied to isolated primary cilia, which showed a strain rate–dependent Young’s modulus in the range of 69.5–240.0 kPa on average. Atomic force microscopy was also performed to measure the local Young’s modulus of primary cilia, showing the Young’s modulus within the order of tens to hundreds of kPa. This study could directly provide the global and local Young’s moduli, which will benefit better understanding of primary cilia mechanics.

## Introduction

Primary cilia are long, thin, microtubule-based organelles protruding from the apical cellular surface (Lim et al., 2015). They are found in multiple types of cells and have been implicated as mechanosensors to sense changes of the surrounding mechanical environment and as chemosensors to detect ligands, growth factors, and hormones. For instance, kidney epithelial cells may use primary cilia to sense urine flow and respond by greatly increasing intracellular calcium (Praetorius and Spring 2001; Praetorius and Spring 2003). It is also reported that endothelial primary cilia may bend in response to blood flow, release calcium, and synthesize nitric oxide (Hierck et al., 2008; Van der Heiden et al., 2008). Furthermore, the lack of primary cilia or their dysfunction may lead to a variety of diseases, such as polycystic kidney disease, blindness, and developmental disorders (Satir et al., 2010; Brown and Witman, 2014; Kowal and Falk, 2015).

To understand the mechanical and biochemical responses of primary cilia to mechanical stimuli, such as fluid flow, many studies have so far been conducted. Schwartz et al. (Schwartz et al., 1997) first modeled a microtubule-based elastic structure of primary cilia to study their bending behavior in response to fluid flow and determine flexural rigidity. However, their model is limited by the assumption of a constant velocity and drag profile along primary cilia. Young et al. (Young et al., 2012) later developed a more precise model of the fluid flow profile around primary cilia and a conducted quantitative comparison of cilia bending between experiments and modeling to obtain flexural rigidity. Downs et al. (Downs et al., 2014) use a coupled fluid-structure interaction model, which combines 3-D fluid dynamics with a large rotation of anchorage of primary cilia, which provides a significantly different flexural rigidity value than the previous studies. In addition to flow experiments, other experimental approaches are employed to study bending characteristics. The flexural rigidity of primary cilia was measured by an optical trap (Battle et al., 2015; Resnick, 2015; Resnick, 2016), which shows that the flexural rigidity of the ciliary axoneme is length-dependent.

Rydholm et al. (Rydholm et al., 2010) structured a finite element model for the apical part of cells, including the primary cilium membrane, to provide flexural rigidity. As for biochemical responses, they also indicate that the delay in calcium response upon bending was caused by the membrane stress at the ciliary base, where the ciliary membrane was modeled continuously with the viscoelastic plasma membrane. In the simulation, the result is strongly influenced by the viscoelastic properties of primary cilia and plasma membranes; however, the viscoelastic properties of primary cilia have never been experimentally studied. As the primary cilium axoneme mainly consist of nine radially arranged microtubule doublets, it is speculated that primary cilia possess viscoelastic properties similar to microtubules (Lin et al., 2007).

As mentioned, most previous researchers study the flexural rigidity of primary cilia, and their results have big discrepancy due to the use of different mathematical models to determine the parameters. However, the material properties of primary cilia, such as Young’s modulus and viscoelasticity, are still not investigated. For a better understanding of primary cilia mechanics, Young’s modulus as well as viscoelastic properties are directly and precisely measured in this study. Recent advances in ultrastructural observation using transmission electron microscopy (TEM) reveal the variations of axoneme configuration from the base to the tip of primary cilia, which has the reduction in the number of microtubule doublets to fewer than nine pairs (Jensen et al., 2004; Gluenz et al., 2010; Sun et al., 2019). Moreover, the TEM observation also reveals that the microtubule doublets exist around a few 10 nm deep from the cilia surface. These changes of structure configuration along the primary cilia suggest a change of mechanical properties along the length of cilia that need the measurement of local Young’s modulus. Atomic force microscopy (AFM) is utilized to determine the local mechanical properties of primary cilia.

In this study, the Young’s modulus of isolated primary cilia is, for the first time, measured by an in-house micro-tensile test (Kojima et al., 1994; Deguchi et al., 2006). Viscoelastic properties of isolated primary cilia are also evaluated by changing the stretching strain rates. Moreover, the local Young’s modulus on the surface of primary cilia is measured to obtain a better picture of the mechanical properties of primary cilia.

## Materials and Methods

### Cell Preparation

Madin–Darby canine kidney cells were used for experiments. Cell culture media consist of Dulbecco’s modified Eagle’s medium supplemented with 10% fetal bovine serum, 1% penicillin, and 1% streptomycin. Cells were cultured under a humidified atmosphere of 5% CO_2_ at 37 °C up to passage 5–10. To carry out the isolation of primary cilia, cells must be confluent and quiescent as primary cilia are formed during the interphase and reabsorbed during mitosis (Plotnikova et al., 2009). Beyond cell confluence, cells continued to grow for 3–5 days to get mature primary cilia and allow them to get their maximal length.

### Isolation of Primary Cilia and Immunofluorescence Staining

For the tensile test, primary cilia were isolated from the cell body using the shear force of rotary shaking (Mitchell, 2013). The isolated cilia were centrifuged at 1,000×g for 10 min at 4°C. The supernatant was transferred to an ultracentrifuge tube and then centrifuged at 40,000×g for 30 min at 4°C. Isolated primary cilia were treated with 0.2% (vol/vol) Triton X-100 for 5 min and then washed with PBS for 10 min. Next, the first antibody (acetylated α-tubulin, Santa Cruz Biotechnology, United States, country) diluted with 1% BSA (1:1,000, Sigma-Aldrich, United States) was added to stain primary cilia for 1 h at 37 °C. Finally, the secondary antibody (Human ads-Alexa Fluor^®^ 488, Southern Biotech, United States) diluted with 1% BSA (1:1,000) was treated for 1 h at 4°C. After each antibody staining step, the solution was centrifuged at 40,000×g for 30 min at 4°C. The pellet containing the primary cilia was transferred to a glass dish, which allows observation under a ×60 lens microscope for the stretching tests.

For the AFM test, another technique to isolate primary cilia was applied (Huang et al., 2006). After removal of the culture medium, cells were washed with PBS three times. A poly-l-lysine–coated coverslip was then placed on the top of the cell monolayer, and PBS was removed with a pipette. A subtle pressure was carefully applied by hand on the top of the coverslip for 20 s. The coverslip was then quickly lifted off with a tweezer. After lifting up, primary cilia stuck to the surface of the coverslip were stained by the aforementioned protocol and used for AFM microscopy.

### Micro-tensile Test

**FIGURE 1:**
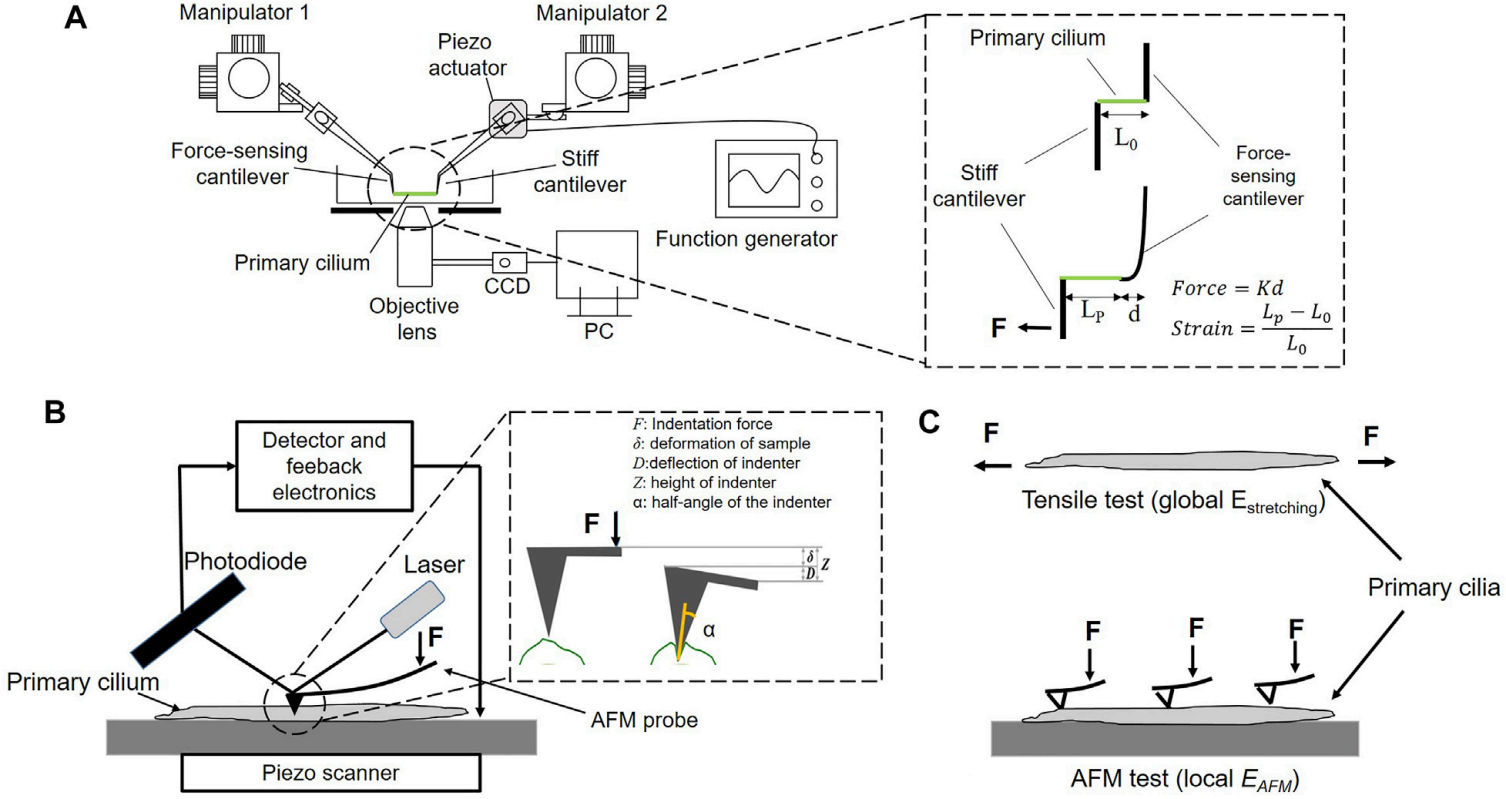
**(A)** Experimental setup of the micro-tensile test. *K*: the spring constant, *d*: the displacement of the force-sensing cantilever, *L*_*0*_ and *L*_*p*_: the length of a primary cilium before and after stretching, respectively **(B)** Experimental setup of the AFM test **(C)** The global Young’s modulus *E*_*stretching*_ and the local Young’s modulus *E*_*AFM*_ of a primary cilium.

A micro-tensile tester was in-house fabricated on an inverted microscope (IX-81, Olympus, Japan) according to the previous study (Deguchi et al., 2006). The stretching test configuration comprises two glass cantilevers, which are controlled by 3-D hydraulic micro-manipulators to place and attach them to both ends of an isolated primary cilium, a piezoelectric actuator (PK2FSF1, Thorlabs, United States), which produces tensile forces to stretch the specimen at different strain rates as shown in Figure 1A. The cantilevers with an appropriate diameter are made from the glass rods (1 mm in diameter) using a glass-electrode puller (Model G1, Narishige, Japan). The spring constant of cantilevers is then determined by a cross-calibration technique. Two types of cantilevers were prepared: a deflectable one with a spring constant of 1–3 nN/μm used as a force sensor and a stiff one connected to the piezo-actuator used to pull the specimen. One end of the stiff cantilevers was thinly coated with an epoxy adhesive (Araldite, Vantico, Japan) and placed on one end of the specimen. The stiff cantilever was then lifted from the substrate and held for 3–5 min until the adhesion hardened. A similar process was conducted to attach one end of the force-sensing cantilever to the other end of the specimen before the stretching test. The stretching test was then performed at 0.01–0.3 s^−1^. It is simply assumed that primary cilia are homogeneous, isotropic, and rounded in cross-section; the Young’s modulus *E*_*stretching*_ is calculated as the following equation:

$$E_{\mathrm{stretching}}=\frac{4\times \frac{\mathrm{dF}}{d\varepsilon }}{\pi D^{2}}$$ (1)

where *F* is an applied force, ε strain, and *D* the diameter of primary cilia.

### Viscoelastic Model

Among the viscoelastic models, the standard linear solid (SLS) model successfully describes both creep and stress relaxation; meanwhile, the other types of model, such as the Maxwell and Kelvin–Voigt models, describe one of those characteristics. In this study, the SLS model was used to simulate the viscoelasticity of primary cilia and perform global fitting with experimental data to determine the viscoelastic parameters. The transfer function *H(s)* in the Laplace domain can be written as follows (Tirella et al., 2014):

$$H\left(s\right)=\frac{\bar{\sigma }}{\bar{\varepsilon }}=E_{0}+\frac{E_{1}\eta_{1}s}{E_{1}+\eta_{1}s}$$ (2)

where *s* is the Laplace operator; $\bar{\sigma }$ and $\bar{\varepsilon }$ stress and strain, respectively, in the Laplace domain; *E*_*0*_ the spring constant in the pure spring arm; *E*_*1*_ the spring constant in the Maxwell arm; and η_1_ the coefficient of viscosity. Finally, stress *σ(t)* in response to an imposed and constant strain rate *ε*_*p*_ is obtained by inverse Laplace transformation as follows:

$$\sigma \left(t\right)=\varepsilon_{p}\left(\eta_{1}-\eta_{1}e^{-\frac{E_{1}t}{\eta_{1}}}+E_{0}t\right)$$ (3)

where *t* is time. To derive the viscoelastic parameters of primary cilia, the data sets of all stress–time series at different strain rates were globally fitted with the mathematical model using Matlab (R2019b, MathWorks, United States).

### AFM

An AFM system (NanoWizard NW3-01H, JPK Instruments, Germany) is equipped with an optical microscope (AXIO observer.A1, ZEISS, Germany). The principle of AFM measurement is schematically shown in Figure 1B. The primary cilium was first identified on the scanning topography image, and then the indenter performed indentation at different points along the cilium to produce the force curve. The Young’s modulus *E*_*AFM*_ was calculated based on the Hertz model as Equation (Hierck et al., 2008), and the tip shape is a conical indenter. In force spectroscopy mode, the contact force can be controlled by changing the applied voltage. To measure the local elastic Young’s modulus *E*_*AFM*_ along the length of primary cilia, voltage of 1 V was applied, producing an indentation depth of 15 nm. Moreover, to study the effect of the indentation depth on *E*_*AFM*_, the applied voltage was set at 0.1, 0.4, 1, and 2 V, which produce a range of indentation depth of 6, 10, 15, and 28 nm, respectively.

$$F=\frac{E_{\mathrm{AFM}}}{1-\nu^{2}}\frac{2\tan\alpha }{\pi }\delta^{2}$$ (4)

where *F* is the contact force, *α* the semi-opening angle of the indenter, *δ* the indentation depth, and ν Poisson’s ratio of sample (it was set to 0.5 in this study). As summarized in Figure 1C, this study employs the global Young’s modulus *E*_*stretching*_ and the local Young’s modulus *E*_*AFM*_ obtained by the stretching test and the AFM test, respectively.

The indentation points were performed equally along the length of the cilium. Due to different lengths of primary cilia, the number of indentation points are different among the cilia. To be uniform for comparison of *E*_*AFM*_, the number of indentation points was downsized to five points in all cilia. For instance, in a case of 10 values of local *E*_*AFM*_ on one cilium, we grouped two values and took the average to get five values of *E*_*AFM*_ on each cilium. The local *E*_*AFM*_ of all cilia are shown in Figure 7.

### TEM

TEM observation was performed to study the detailed structure of primary cilia on cells. Cells were subjected to prefixation in 2% glutaraldehyde in 0.1 M phosphate buffer (pH 7.4) for 30 min and then postfixation with 1% osmium tetroxide in 0.1 M phosphate buffer (pH 7.4) for 2 h. After rinsing extensively, cells were transferred into a resin block. The resin blocks were sectioned at 70 nm, collected onto copper grids, and stained with 2% uranyl acetate for 10 min and photographed under TEM (JEM 2100, JEOL, Japan).

### Immunofluorescence Staining of Primary Cilia on Cells, Actin Filaments, and Nuclei

Immunolabeling of acetylated α-tubulin was performed to visualize primary cilia on cells under the fluorescence microscope. After rinsing in PBS, cells were fixed with 4% paraformaldehyde for 30 min at room temperature, treated with 0.2% (vol/vol) Triton X-100 in PBS for 5 min, and blocked with 1% BSA blocking solution. Cells were stained with the first antibody (acetylated α-tubulin, Santa Cruz Biotechnology, United States) diluted with 1% BSA (1:1,000, Sigma-Aldrich, United States) overnight at 4°C and then in secondary antibody (Human ads-Alexa Fluor^®^ 488, Southern Biotech, United States) diluted with 1% BSA (1:1,000) at 4°C for 1 h. Subsequently, the actin filaments and nuclei were stained with 400X Rhodamine Phalloidins (ThermoFisher, United States) diluted in PBS (1:400) for 1 h and Hoechst 33,342 (ThermoFisher, United States) for 20 min, respectively.

### Statistical Analysis

Results are shown as mean ± SD. Statistical significance was determined using Student’s *t*-test. For all tests, *p* < 0.05 was considered significant.

## Results

### Visualization of Primary Cilia

**FIGURE 2:**
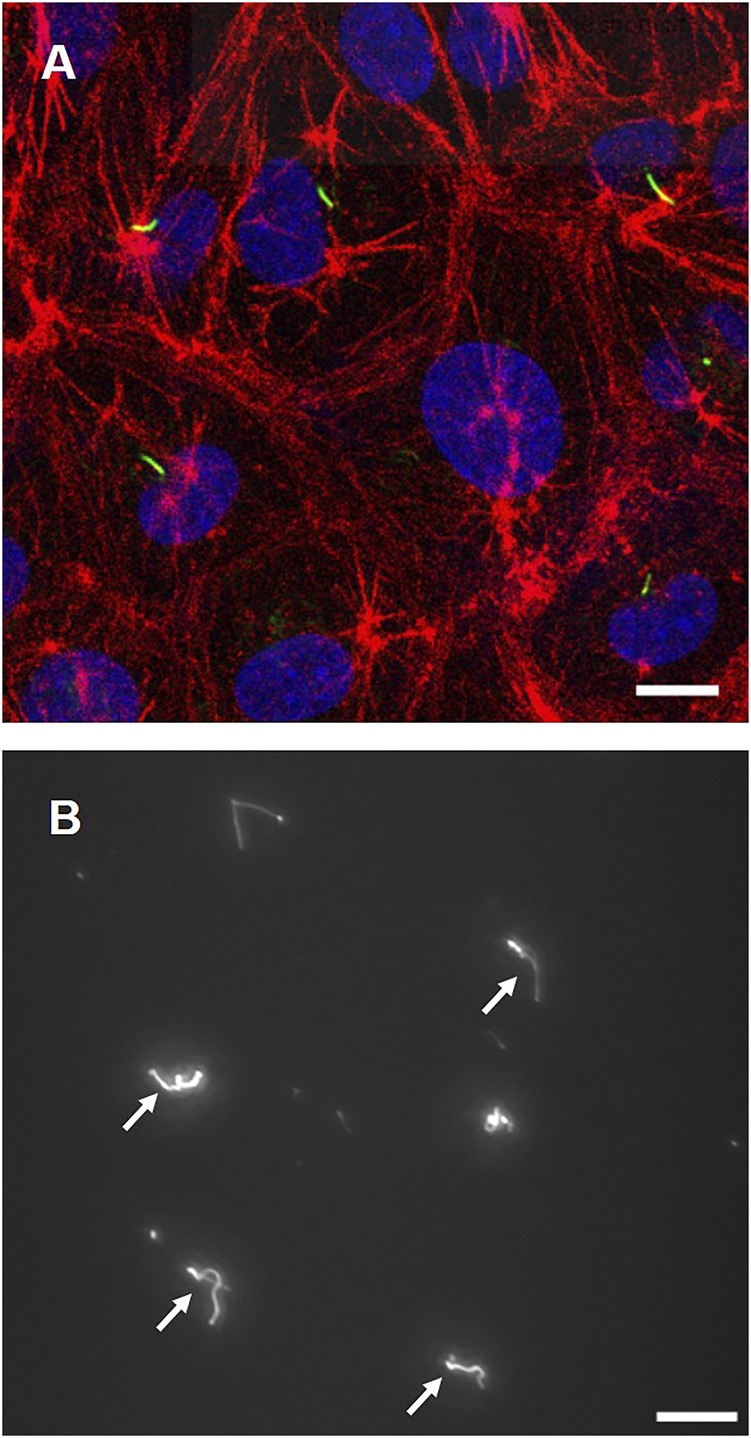
**(A)** Cell images with primary cilia (green), actin filaments (red), and nuclei (blue) **(B)** Isolated primary cilia after ultracentrifugation (white arrows). Scale bar: 10 µm.

Figure 2A shows fluorescence images of primary cilia (green), actin filaments (red), and nuclei (blue). The incidence of primary cilia is around 52%. Cells exhibit a relatively rounded shape with dense peripheral bands of actin filaments at the cell peripheries. Figure 2B shows primary cilia isolated from cells. Primary cilia were successfully isolated and collected through the ultracentrifuge protocol.

### TEM Observation

**FIGURE 3:**
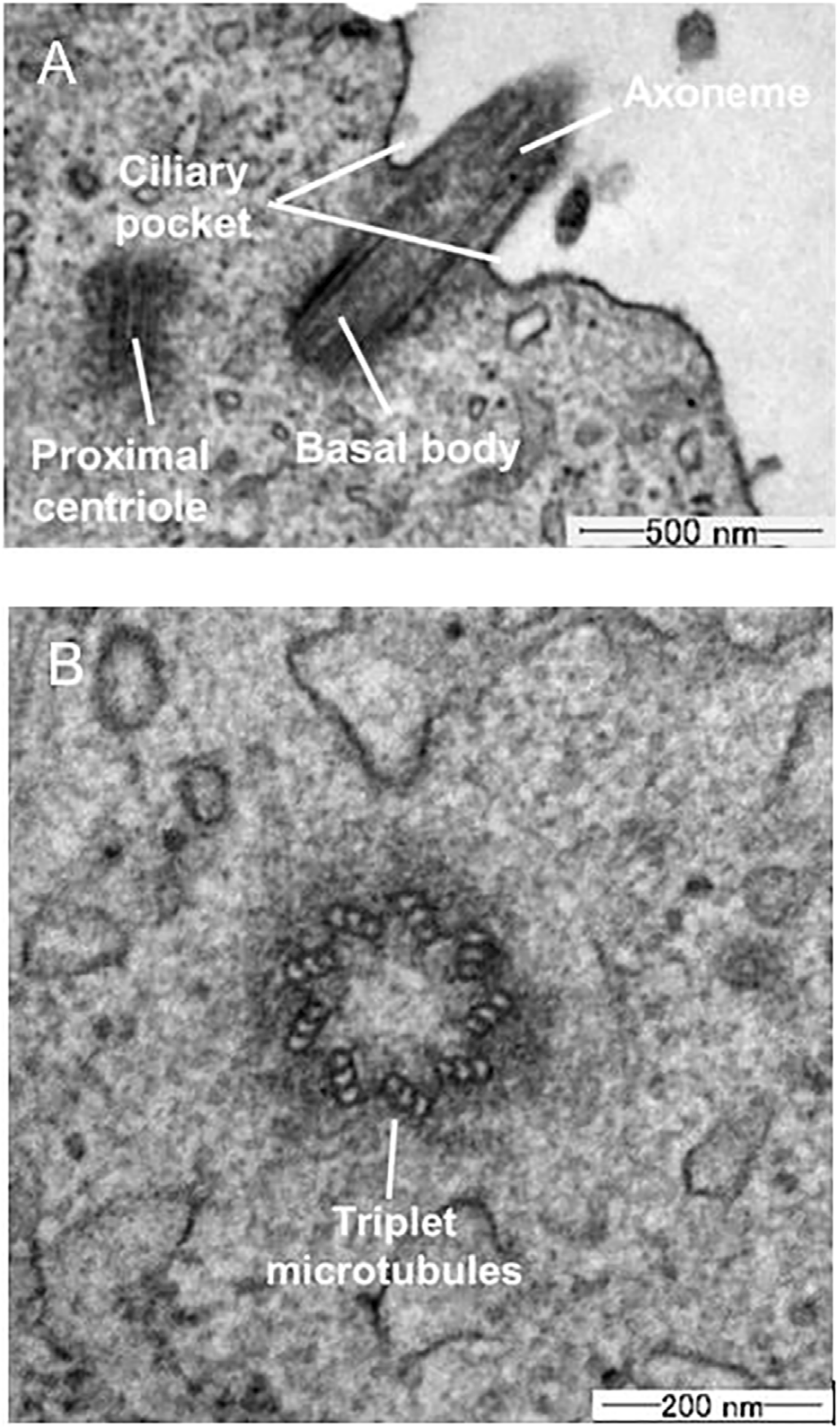
TEM images **(A)** Longitudinal section **(B)** Cross-section of primary cilia.

Figure 3A shows a TEM image of the microstructure components of a primary cilium. In the axoneme, doublet microtubule structures are running through the axis. Figure 3B shows a cross-sectional image of a primary cilium. Triplet microtubules at the base of the cilia are clearly observed in the section, in which nine doublet microtubules are continuously projected to the membrane. Based on the cross-sectional image, the diameter of primary cilia was measured and determined to be 205.2 ± 30 nm (mean ± SD, *n* = 4), which is similar to the previously reported diameter of ca. 0.2 µm (Jensen et al., 2004; Rydholm et al., 2010; Jin et al., 2014).

### Young’s Modulus and Viscoelastic Properties

**FIGURE 4:**
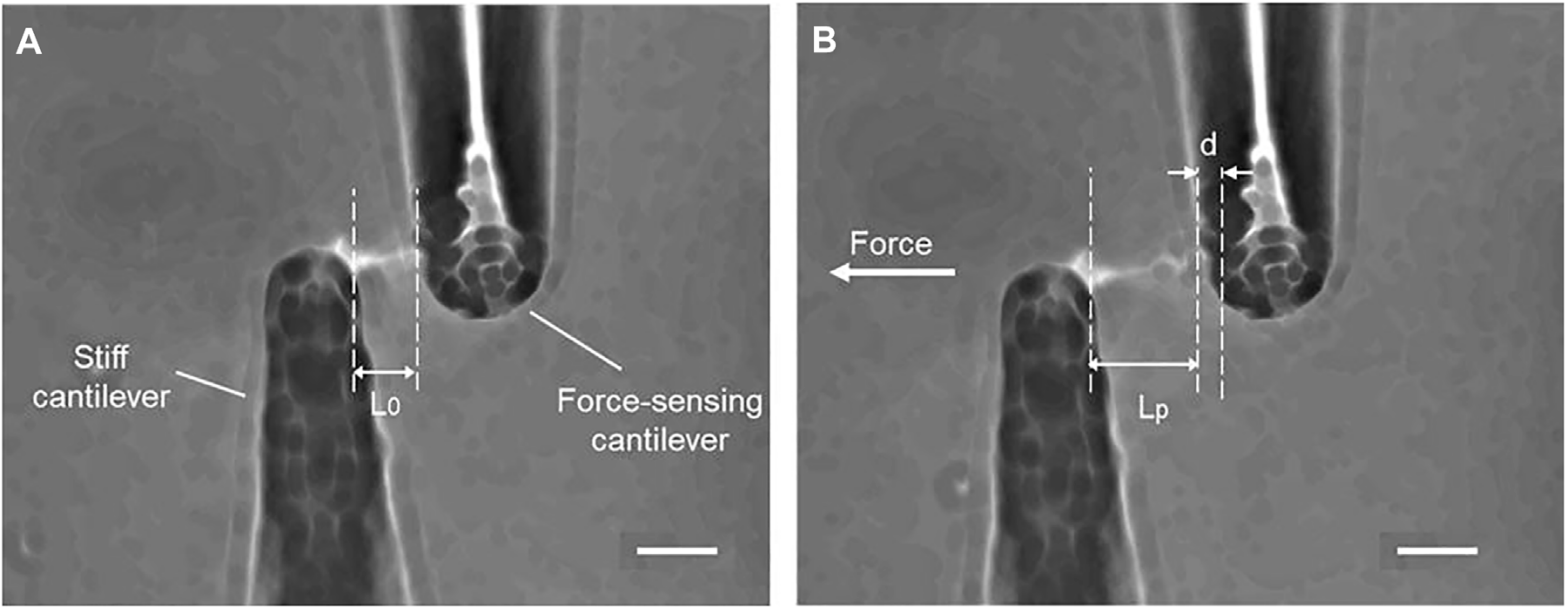
Tensile stretching of primary cilia **(A)** Before stretching **(B)** During stretching, *d*: the displacement of the force-sensing cantilever, *L*_*0*_ and *L*_*p*_: the length of a primary cilium before and after stretching. Scale bar: 10 µm.

**FIGURE 5:**
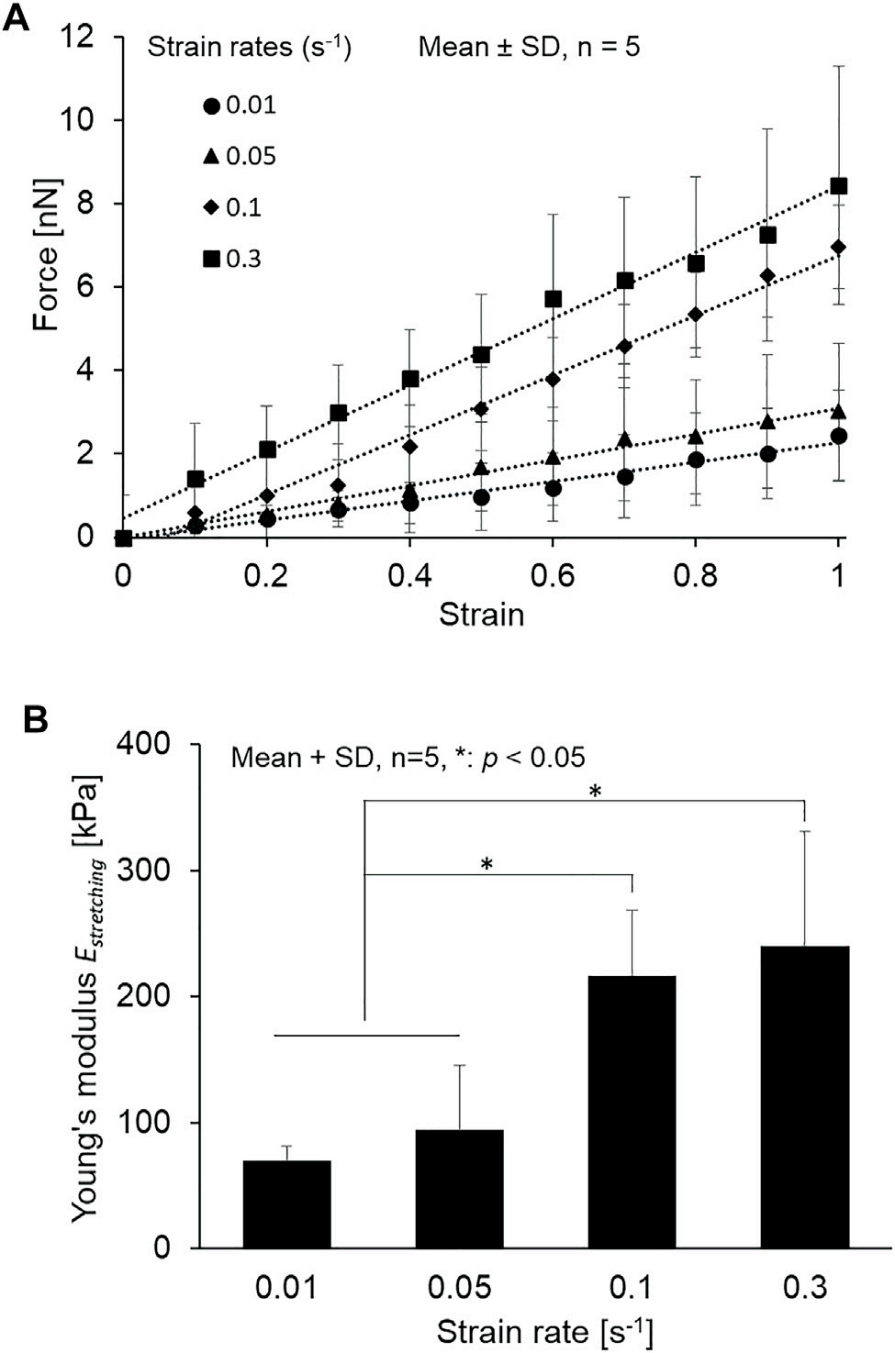
**(A)** Relationship between force and strain of primary cilia at different strain rates from 0.01 to 0.3 s^−1^ and the least squares fitting **(B)** The Young’s modulus determined at different strain rates from 0.01 to 0.3 s^−1^.

As shown in Figure 4, the cilia are stretching after applying tensile force, and the small displacement of a force-sensing cantilever is indicated. The force–strain relationships at the different strain rates of 0.01–0.3 s^−1^ are summarized in Figure 5A. Primary cilia linearly elongate with increasing applied force within this strain range. The least squares fitting to the experimental data was applied to determine the global Young’s modulus *E*_*stretching*_, showing 69.5 ± 12.1, 94.1 ± 51.4, 216.7 ± 51.4, and 240.0 ± 90.7 kPa for strain rates of 0.01, 0.05, 0.1, and 0.3, respectively, as shown in Figure 5B. The Young’s moduli at the strain rates of 0.1 and 0.3 are significantly higher than those at 0.01 and 0.05, indicating the strain rate–dependence of viscoelastic properties of primary cilia.

**FIGURE 6:**
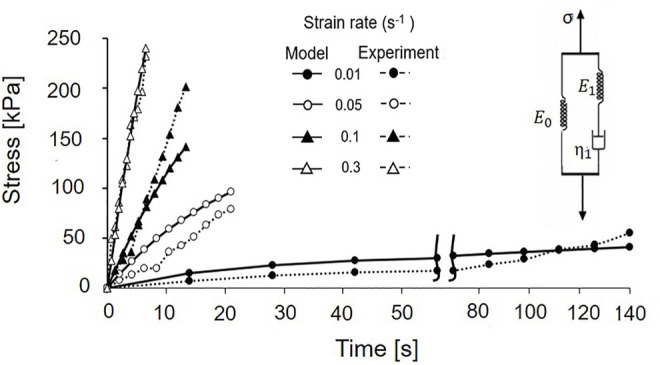
Global fitting of the SLS model and experimental data sets.

Figure 6 shows the change in stress of isolated primary cilia over time and the global fitting of the Maxwell-type SLS model to the experimental data sets. The instantaneous elastic moduli *E*_*inst*_ = *E*_*0*_ + *E*_*1*_, the equilibrium elastic moduli *E*_*eq*_ = *E*_*0*_, and the relaxation time τ_1_ = *η*_*1*_ */E*_*1*_ of the SLS model are summarized in Table 1 with the coefficient determination *R* ^2^ = 0.86.

**TABLE 1:** Viscoelastic parameters of primary cilia derived from fitting experimental data and the Maxwell-type SLS model of primary cilia.

Viscoelastic parameters	Values
*E*_*inst*_ = *E*_*0*_ *+ E*_*1*_ [kPa]	143.2 ± 3.0
*E*_*eq*_ = *E*_*0*_ [kPa]	11.0 ± 3.0
*τ*_*1*_ = *η*_*1*_ */E*_*1*_ [s]	19.36 ± 3.7
*R* ^2^	0.86

### AFM Measurement

**FIGURE 7:**
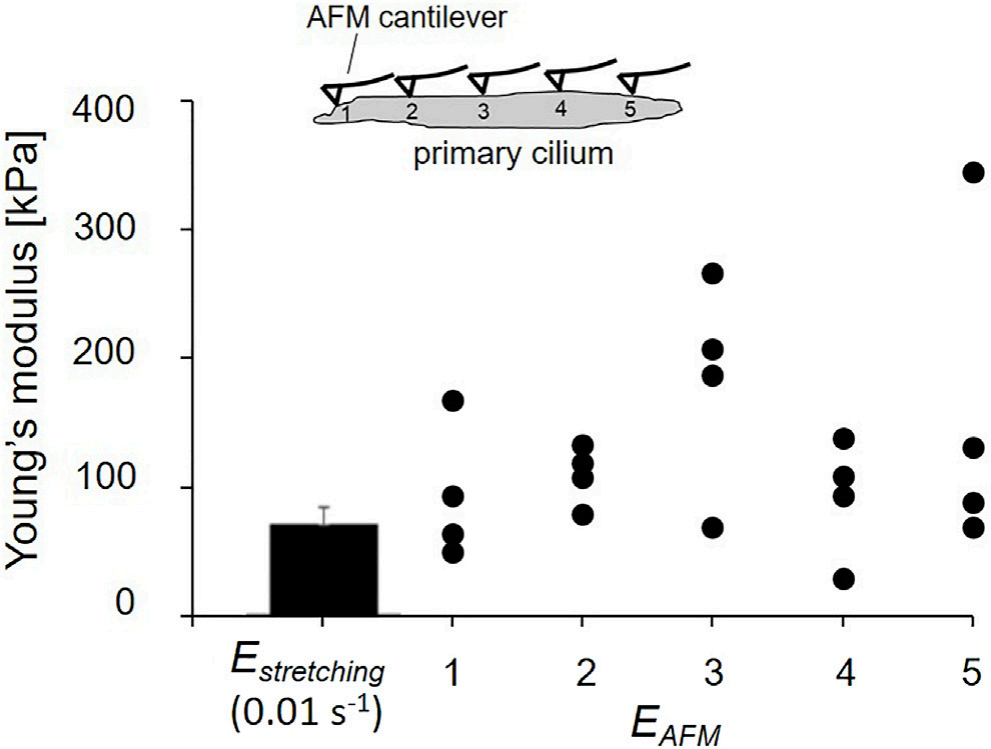
Comparison of global *E*_*stretching*_ and local *E*_*AFM*_ *.*

Figure 7 shows the distribution of local Young’s modulus *E*_*AFM*_ along the length of tested cilia. The global Young’s modulus *E*_*stretching*_ in the static condition (0.01 s^−1^) is the same order as the local *E*_*AFM*_ values. This agreement between global *E*_*stretching*_ and local *E*_*AFM*_ shows the confidence of the obtained elastic properties of primary cilia.

**FIGURE 8:**
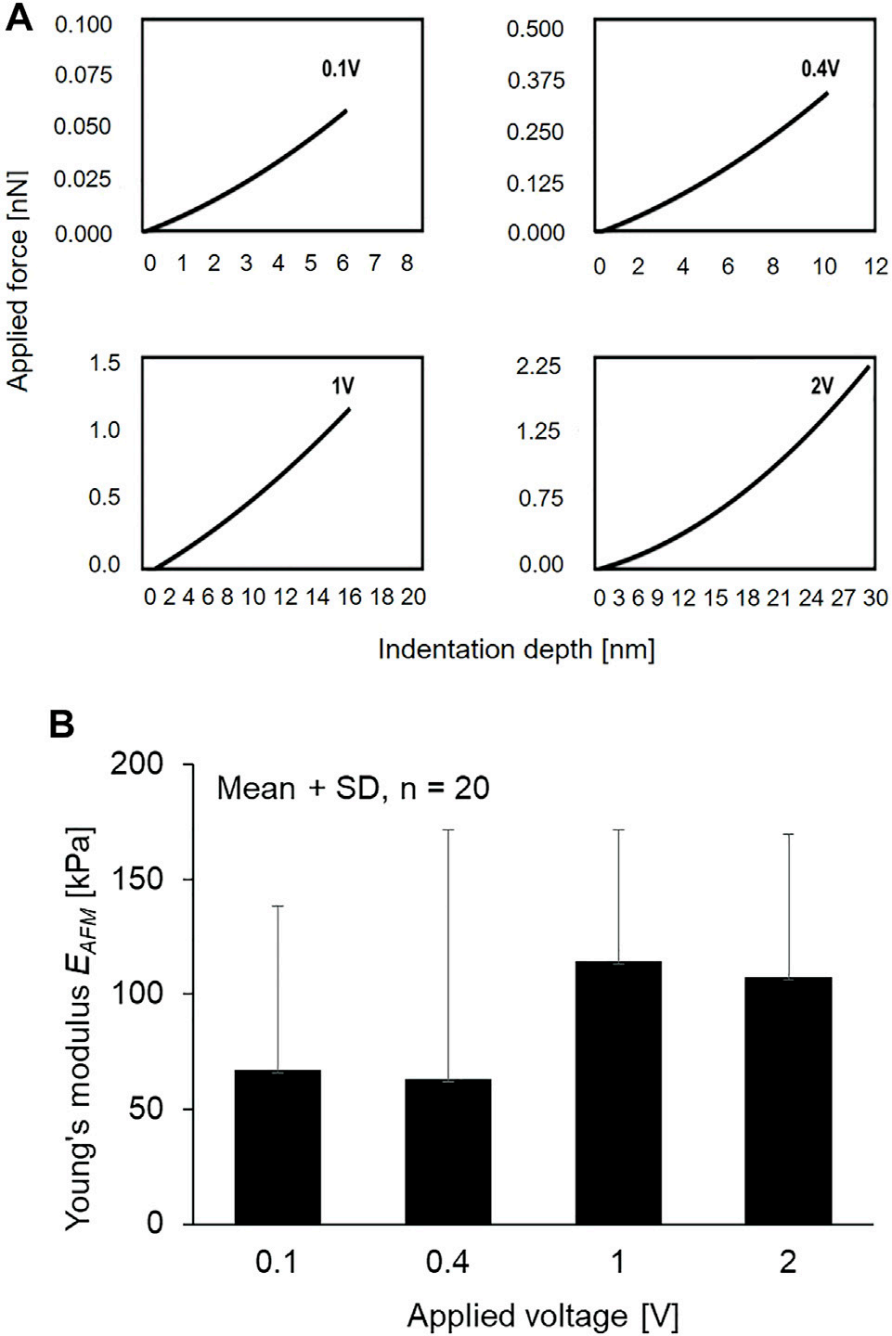
**(A)** Relationship of applied voltage and indentation depth **(B)** The Young’s modulus of primary cilia at different applied voltage.

Figure 8A shows a representative contact force–indentation depth curve at an arbitrary position with the different applied voltages. This result indicates that the applied voltage of 0.1, 0.4, 1, and 2 V can approximately generate indentation depths of 6, 10, 15, and 30 nm, respectively. The local Young’s modulus *E*_*AFM*_ of the primary cilia was determined with the different applied voltages as shown in Figure 8B. With increasing the applied voltage, the local Young’s modulus *E*_*AFM*_, in particular, became higher from 10 to 15 nm although there are no significant differences between the experimental groups.

## Discussion

To date, the flexural rigidity of primary cilia has been extensively studied mainly by application of fluid flow because of the many advantages of such an experimental approach: quick setup of experimental apparatus, *in vivo* relevance, etc. However, flexural rigidity is not a material property of materials, but depends on the shape and size of the cross-section of specimens. Moreover, a mathematical model has to be introduced to determine the flexural rigidity, which could potentially produce a discrepancy between literatures. To our best knowledge, this study is the first report that the Young’s modulus of primary cilia can be directly measured. Schwartz et al. (Schwartz et al., 1997) develops the first model of the bending mechanics of kidney epithelial primary cilia, observes bending of primary cilia under various physiological fluid flow rates, and determines the flexural rigidity to be approximately 3.1 × 10^−23^ N m^2^. Later, other groups determined the values to be in the range of 1–5 × 10^−23^ N m^2^ (Young et al., 2012) or even significantly higher values of 3.1 × 10^−22^ N m^2^ (Downs et al., 2014). The global Young’s modulus *E*_*stretching*_ statically obtained at a strain rate of 0.01 s^−1^, as shown in Figure 5B, is 69.5 ± 12.1 kPa. To directly compare our study with the previous studies, the flexural rigidity was converted into the Young’s modulus under the assumption that primary cilia have a uniform rounded-shape cross-section with a diameter of 205 nm (obtained in this study) by using Equation 5.

$$E=\frac{\mathrm{EI}}{I}=\frac{\mathrm{EI}}{\left(\frac{\pi a^{4}}{4}\right)}$$ (5)

where *E* is Young’s modulus, *EI* the flexural rigidity, and *a* the radius. The comparison is summarized in Table 2. Except for Downs’s group, the Young’s modulus *E*_*stretching*_ in this study shows similar values to the others although there are some discrepancies between the results and our value being the smallest. As mentioned in the Introduction, previous studies use different mathematical models to determine the flexural rigidity. Moreover, as the diameter of 205 nm that is experimentally determined in this study is used for conversion, it is quite possible that the actual diameter of primary cilia used in the previous studies are different from 205 nm. The discrepancies may inherently be attributable to these facts. The unique aspect of this study compared with the previous studies is that the Young’s modulus of primary cilia is directly measured, in other words, not affected by the use of mathematical models, and it does not need any conversion.

**TABLE 2:** Summary of the Young’s modulus of primary cilia.

	Author, year (ref)	Flexural rigidity [Nm^2^]	Young’s modulus [kPa]	Experimental method
Primary cilia	Schwartz et al. (1997)	3.1 × 10^−23^	356.9 (converted)	Fluid flow
	Young et al. (2012)	1–5 x 10^−23^	115.1–575.7 (converted)	Fluid flow
	Downs et al. (2014)	31 × 10^−23^	3,569 (converted)	Fluid flow
	Battle et al. (2015)	2.5 × 10^−23^	287.9 (converted)	Optical trap
	Resnick (2016)	1.7 × 10^−23^	199.2 (converted)	Optical trap
	Present study	—	143.2	Tensile test
Microtubules	Tuszyński et al. (2005)	—	1.32 × 10^6^	Tensile test
	Gittes et al. (1993)	2.2 × 10^−23^	—	Thermal bending
	Kikumoto et al. (2006)	7 × 10^−23^	—	Optical trap

Primary cilia are microtubule-based organelles, and the core axoneme comprises nine doublet microtubules. The Young’s modulus (Tuszyński et al., 2005) and flexural rigidity (Gittes et al., 1993; Kikumoto et al., 2006) of microtubules are reported in Table 2. The Young’s modulus is on the order of GPa; however, the Young’s modulus of primary cilia obtained in this study is on the order of tens to hundreds of kPa. This discrepancy in the Young’s modulus between microtubule-based primary cilia and microtubules can possibly be explained based on the structure. In nonmotile cilia, such as primary cilia, it is not clear whether there are structural components, such as the dynein arms, to connect the double microtubules together. During the tensile process, the dynein arms pull double microtubules away from each other and may mainly contribute the mechanical properties of motile cilia. The results here may suggest alternative components, similar to dynein arms, which maintain the axoneme’s stability and elastic properties for reversible bending of primary cilia. Further investigations could focus on clarifying these components in the structure of primary cilia.

There is no study so far about the viscoelasticity of primary cilia. Our global fitting between the experimental data and mathematical model (Figure 6), for the first time, provides the viscoelastic parameters of primary cilia. The instantaneous elastic modulus is on the same order as the converted Young’s modulus in Table 2. Regarding the relaxation time, it has a high diversity of values from previous studies. Young et al. (Young et al., 2012) reports the relaxation of the cilium after turning off the application of flow was around 5 s; meanwhile, that value for Downs et al. (Downs et al., 2014) was around 2 min. This discrepancy may arise from a difference in fluid flow conditions. It is unlikely to compare the relaxation time of isolated cilia and cell-attached cilia under fluid flow because of taking into account the rotational relaxation of basal body anchorage. Compared with the relaxation of the microtubule, which is reported to be around 600 s (Lin et al., 2007), the relaxation time of primary cilia is much smaller. It can be explained by the fact that, besides the nine doublet microtubules, there are other components inside the cilia structure to couple microtubules together and contribute to the mechanical properties of primary cilia.

In Figure 7, the SD of the Young’s modulus *E*_*stretching*_ is small, which is partly because *E*_*stretching*_ is an averaged modulus across not only the cross-section, but also the length. In contrast, the figure clearly indicates the wide range of distribution in local Young’s modulus *E*_*AFM*_ along the length of the primary cilia. Primary cilia structure nonuniformly along their length because nine doublet microtubules run in parallel through their length and the number of doublet microtubules decreases from the base to the ciliary tip (Gluenz et al., 2010) and are, thus, considered to be inhomogeneous at the nanoscale. Different positions of indentation may reach different structures of primary cilia, possibly with or without touching the doublet microtubules, which could lead to the wide range of *E*_*AFM*_ distribution. The measurement of *E*_*AFM*_ opens a new topic for further studies of primary cilia mechanics. For instance, the local distribution of the Young’s modulus may contribute to building up more precise mathematical models to predict the bending behavior of primary cilia. The combination of the micro-tensile and AFM tests allows us to obtain more clearly insight into the mechanical properties of primary cilia.

As shown in Figure 8, the local Young’s modulus *E*_*AFM*_ became higher when the indentation depth increased from 0.4 to 1 V. It is known from TEM observation that the double microtubules distribute evenly surrounding the centerline of the ciliary body with a distance of 0.07 µm to the center, and double microtubules exists around several 10 nm deep from the cilia surface (Chen et al., 2011). The increase in Young’s modulus may be reflected by the presence of the doublet microtubules where the AFM tip more directly comes into contact with the structure. Because of the high Young’s modulus of microtubules, it is assumed that the local Young’s modulus *E*_*AFM*_ was higher when the indenter could reach the double microtubules.

This study has certain limitations. Regarding the local Young’s modulus *E*_*AFM*_ along the length of the cilia, isolated primary cilia could not be distinguished correctly between the base and the ciliary tip, which limits the evaluation of the actual distribution of the local Young’s modulus. The base of the primary cilia comprises a full nine doublet microtubules, and the doublet microtubule number decreases toward the tip of the cilia suggesting different mechanical properties along the length of the cilia. In addition, it should be noted that, because microtubules form a radial array of nine doublets, there is a possibility that the AFM tips indent the surface where the doublet microtubules do not exist right under the position. This possibility may be involved in the large SD in the results. In the global fitting method, despite the good agreement of Young’s modulus of primary cilia with previous converted results (Table 2), the *R* ^2^ value of 0.86 and the not-good fitting between strain rate groups can be seen. The fast strain rate groups have better fitting than the lower groups. It is possible that the fitting results could be more precisely identified depending on the number of strain rates used and the range of strain rates employed. The higher number of strain rates and the smaller gap between strain rates may establish better parameters of Young’s modulus and relaxation time.

As mentioned, numerous efforts have so far been made to characterize the bending behavior of primary cilia; however, they seem to lack the validation of material properties, such as Young’s modulus. It would be beneficial to directly measure Young’s modulus from the viewpoint of cell mechanics. A further approach would be needed to shed light on how the global and local Young’s moduli could attribute to their structure. For instance, nine doublet microtubules are connected by dynein arms in motile cilia although it is unclear if there are alternative structural components integrating the nine microtubule in nonmotile cilia, such as primary cilia. Such an experimental approach based on the microstructure would lead to better understanding of primary cilia mechanics as well as mechanotransduction.

## Conclusion

In this study, the tensile Young’s modulus of primary cilia was, for the first time, measured with an in-house micro-tensile tester. The tensile stretching at different strain rates clearly indicates the viscoelastic properties of primary cilia, which would be attributable to the microtubule-based structure. Moreover, the local Young’s modulus on primary cilia was also measured by using the AFM test, which may possibly be reflected by the presence of doublet microtubule structures. These results may help to have a better picture of the mechanotransduction of primary cilia in further experimental studies and may contribute to building up an appropriate cellular model in numerical studies.

## Data Availability

The original contributions presented in the study are included in the article/Supplementary Material, further inquiries can be directed to the corresponding author.
